# Trimetazidine offers myocardial protection in elderly coronary artery disease patients undergoing non-cardiac surgery: a randomized, double-blind, placebo-controlled trial

**DOI:** 10.1186/s12872-021-02287-w

**Published:** 2021-10-01

**Authors:** Zhong-Liang Dai, Yi-Feng Song, Ya Tian, Yin Li, Miao Lin, Juan Lin, Qi Wang, Ping Wang, Wen-Li Gao

**Affiliations:** 1 Department of Anesthesiology, Shenzhen People’s Hospital (The Second Clinical Medical College, Jinan University; The First Affiliated Hospital, Southern University of Science and Technology), Shenzhen, 518020 Guangdong China; 2 Shenzhen Engineering Research Center of Anesthesiology, No. 1017 Dongmen North Road, Shenzhen, 518020 Guangdong China

**Keywords:** Trimetazidine, Elderly, Surgery, Myocardial ischaemia, Complications

## Abstract

**Background:**

Trimetazidine (TMZ) pretreatment protects cardiomyocytes during cardiac surgery. TMZ may protect elderly patients with ischaemic heart disease (IHD) undergoing non-cardiac surgery.

**Methods:**

This was a randomized, double-blind, placebo-controlled trial (registration #ChiCTR1900025018) of patients with IHD scheduled to undergo non-cardiac surgery at Shenzhen People’s Hospital (Shenzhen, Guangdong Province, China) between June 2014 and September 2015, randomized to 60 mg TMZ or placebo 12 h before surgery. The primary endpoint was the occurrence of in-hospital cardiovascular events. The secondary endpoints were myocardial ischaemia on five-lead electrocardiogram (cECG), cardiac troponin I (cTnI) elevation, cardiac death, acute coronary events, heart failure, and arrhythmia requiring treatments.

**Results:**

Compared with the placebo group, the TMZ group showed a lower occurrence of in-hospital cardiovascular events (primary endpoint, 20.0% vs. 37.5%, *P* = 0.02), myocardial ischaemia (15.0% vs. 32.5%, *P* < 0.01), cTnI elevation (2.5% vs. 10%, *P* < 0.01), acute coronary events (10.0% vs. 20.0%, *P* < 0.05), heart failure (0% vs. 2.5%, *P* < 0.05), and arrhythmia requiring treatment (17.5% vs. 35.0%, *P* < 0.05). There was no acute myocardial infarction during the 30-day postoperative period.

**Conclusions:**

In elderly patients with IHD undergoing non-cardiac surgery, TMZ pretreatment was associated with myocardial protective effects.

*Trial registration* The trial was prospectively registered at http://www.chictr.org.cn/showproj.aspx?proj=41909 with registration number [ChiCTR1900025018] (7/8/2019).

## Background

The prevalence of heart disease, especially acute myocardial infarction, has increased in the past ten years [1]. The proportion of the population over 65 years of age is increasing at a much faster rate than the population growth in general [2]. A history of ischaemic heart disease (IHD) is a risk factor for postoperative cardiac events, and the incidence of clinically significant cardiac events after non-cardiac surgery is as high as 2.43% [3]. For those with IHD, the perioperative complications of non-cardiac surgery vary from arrhythmias to heart failure [4]. Anaesthesiologists will inevitably have to participate in elective or emergency operations in elderly patients with either confirmed or suspected IHD. Many patients experiencing perioperative myocardial injury are asymptomatic. Perioperative myocardial injury increases 30-day mortality by nearly tenfold [5]. Therefore, these patients are in need of safety and efficacy measures to prevent myocardial injury.

Mounting evidence suggests that IHD is highly manageable [6, 7]. The key is to balance the myocardial oxygen demand and supply [8]. Trimetazidine (1-[2,3,4-trimethoxybenzyl] piperazine dihydrochloride; TMZ) is a well-known anti-ischaemic agent used in myocardial protection [9]. Preoperative TMZ therapy appears to have a positive effect on myocardial preservation in patients undergoing coronary artery bypass graft (CABG) [10]. In addition, TMZ has been used in metabolic therapy in recent years to optimize the myocardial use of energy [11]. Most studies have focused on the molecular mechanism. Zhong et al. [12] discovered that TMZ protects against ischaemia/reperfusion (I/R) injury by improving autophagic flux through the AMPK signalling pathway. Liu et al. [13] discovered that TMZ inhibits coronary microembolization-induced myocardial apoptosis. Danikiewicz et al. [14] showed that TMZ treatment led to a decrease in IL-2 and IL-8 levels before a treadmill exercise test. The Nrf2/NF-κB pathway in cardiomyocytes may be a possible mechanism of the abovementioned effects of TMZ [15].

Although the cardioprotective effect of TMZ has been well acknowledged [16, 17], it remains unknown whether these protective effects can be observed during non-cardiac surgery. Therefore, the aim of the present trial was to confirm the protective effects of TMZ in elderly patients with IHD undergoing non-cardiac surgery.

## Methods

### Study design and patients

This was a randomized, double-blind, placebo-controlled study approved by the Shenzhen People's Hospital Ethics Committee, Guangdong, China (Chairperson Prof MJ. Tang, Ethics Committee Number LL-KT-2014028). All subjects were properly informed of the risks and procedures of the research before enrolment. All participants provided written informed consent. This clinical trial has been registered (#ChiCTR1900025018). All methods were performed in accordance with the relevant guidelines and regulations.

The inclusion criteria were as follows: male or female patients (1) > 60 years of age; (2) diagnosed with IHD according to the diagnostic criteria of coronary artery disease published by the American Heart Association [18]; (3) with American Society of Anesthesiologists (ASA) grade II–IV; (4) willing to participate and to complete the trial; and (5) scheduled for major non-cardiac surgery such as vascular procedures of the abdominal aorta or the lower limb, open intraperitoneal or intrathoracic procedures, and major orthopaedic procedures of the hip or spinal column, among others.

The exclusion criteria were as follows: (1) congenital heart disease, rheumatic heart disease, cardiomyopathy, left ventricular hypertrophy, or valvular disease; (2) history of myocardial infarction in the previous 3 months; (3) mental disorders; and (4) any history of TMZ use.

### Randomization and blinding

Randomization was carried out using a random number table prepared by an independent statistician using SPSS 13.0 (SPSS Inc., Chicago, IL, USA). The randomization sequence was prepared in sealed envelopes. On the day before surgery, an envelope was randomly taken to the ward by one of the investigators. Concealment was maintained until 12 h before induction, when a nurse opened the envelope. Patients, data collectors, and data analysts were blinded to the type of drug used. On the day before surgery, both TMZ and placebo (of the same colour, size, and amount; 20 mg/tablet) were delivered to the ward nurse in charge of the patient. The preparation and dispensing of the tablets were performed by the ward nurse. The investigators were blind to the treatment allocation.

### Medication, anaesthesia, and monitoring

All patients fasted for 8–12 h before surgery. There was no premedication. Concurrent medications, such as β-blockers, ACEIs, aspirin, and statins, were continued through the morning of surgery. According to the package insert and previous references [9–11], twelve hours before induction, the patients took the tablets orally prepared by the nurse (60 mg, three tablets, 20 mg/tablet). As soon as the patient arrived in the operating room, standard monitoring started with 5-lead electrocardiogram, pulse oximetry, non-invasive blood pressure, invasive radial arterial pressure (performed only after Allen’s test was negative), inspiratory and expiratory gas concentrations, and electroencephalography monitoring (bispectral index monitor, Covidien, Dublin, Ireland). Ringer’s solution was given at a dose of 4 ml/kg as a preload.

All patients received intravenous/inhalation combined anaesthesia. Anaesthesia was induced with midazolam 0.02 mg/kg, etomidate 0.3 mg/kg, cisatracurium 0.25 mg/kg, and fentanyl citrate 3 µg/kg. As soon as the eyelid reflex was absent, assisted ventilation by facemask was started with 100% oxygen. After 5 min of denitrogenation, orotracheal intubation was performed (the size of the catheter was 7.5# for males and 7.0# for females). Ventilator parameters (tidal volume 5–6 ml/kg, breath rate 12–16/min) were adjusted so that the end-tidal carbon dioxide (ETCO_2_) was maintained between 35 and 45 mmHg. During surgery, anaesthesia was maintained with sevoflurane, propofol 2 mg/kg/h, remifentanil 0.15 µg/kg/min, and a bolus dose of cisatracurium 0.03 mg/kg if needed. The fluid volume was maintained with Ringer’s solution and hydroxyethyl starch. The depth of anaesthesia was adjusted according to vital signs and the bispectral index (BIS) so that the optimal index was maintained between 45 and 60. Mean blood pressure and heart rate were maintained at ± 20% of baseline. Sevoflurane was discontinued 15 min before the end of surgery, and the oxygen flow was increased to 5 l/min to refresh the lung, while the dose of propofol was increased to maintain an appropriate depth of anaesthesia. As the surgery finished, all drugs were ceased, and intravenous analgesia was started. Secretions in the trachea and mouth were suctioned. Extubation was performed when the swallowing reflex and spontaneous breathing recovered, and the patient was able to respond. After removing the radial artery catheter, the patient was sent back to the ward.

### Blood samples

Blood samples were collected before and after surgery, including 1 ml of arterial blood for blood gas analysis and 3 ml of venous blood. Venous blood (3 ml) was also collected 24 and 48 h after surgery.

### Data collection

The preoperative data collection included sex, age, body mass index (BMI), the Lee Revised Cardiac Risk Index, the degree and extent of coronary artery disease, NYHA classification, ASA classification, and preoperative medications. The intraoperative data collected included the type of surgery, amount of blood loss, duration of operation, mean inspired sevoflurane concentration, mean propofol infusion rate, mean total remifentanil given perioperatively, and BIS values. The postoperative data were myocardial ischaemia on five-lead electrocardiogram (ECG); occurrence of acute myocardial infarction (AMI); clinically relevant arrhythmias, including atrial fibrillation and bundle branch block, ventricular tachycardia, ventricular fibrillation, or any arrhythmia that affect blood pressure and requiring treatment; length of ICU stay; length of stay (LOS) in hospital; reoperation within the hospital stay; and any adverse event not otherwise specified. The diagnosis of AMI was based on a rise in cardiac troponin I (cTnI) to > 0.3 ng/ml postoperatively and at least one of the following criteria: (1) symptoms of ischaemia; (2) new electrocardiogram changes indicative of ischaemia (ST and/or T changes or left bundle branch block presumed to be of recent onset); and (3) development of pathological Q waves in the electrocardiogram. ECGs were obtained by an independent investigator not involved in the treatment of the patient. At 30 days after surgery, data about mortality, AMI, arrhythmias, cardiac dysfunction, readmissions, and any kind of adverse event not otherwise specified were collected. A telephone interview was conducted with the patients and their families. All data were collected by researchers who did not participate in patient care.

### Endpoints

The primary endpoint was the occurrence of in-hospital cardiovascular events. In-hospital ‘cardiovascular events’ included AMI, new or presumed new significant ST-segment–T wave (ST–T) changes or new left bundle branch block (LBBB), development of pathological Q waves in the ECG, imaging evidence of new loss of viable myocardium and any new regional wall motion abnormality identified as an intracoronary thrombus by angiography or autopsy. The secondary endpoints were myocardial ischaemia on cECG, cTnI elevation (> 0.3 ng/mL), cardiac death, acute coronary events, heart failure (left ventricular ejection fraction less than 40%), and arrhythmia requiring treatment.

### Sample size

We used the occurrence of in-hospital cardiovascular events for sample size calculation. A 20% difference (20% and 40%) between the two groups was regarded as clinically relevant on the basis of other studies [19–21]. Using an α error of 5% and a test power of 80%, power analysis indicated that such a difference would be detected with a sample size of n = 60 (or 30 per group). Assuming a loss to follow-up of 20%, each group had to have 40 patients at enrolment.

### Statistical analysis

Analyses were performed using SPSS 13.0 (SPSS Inc., Chicago, IL, USA). Continuous data were tested for normality of their distribution using the Kolmogorov–Smirnov test. Continuous data are reported as mean ± SD or as medians (interquartile range). Differences between groups were assessed using Student’s t-test or the Mann–Whitney U test, according to distribution. Categories are presented as percentages and were compared using the chi-square test or Fisher’s exact test, as appropriate. Significance was set at *P* < 0.05.

## Results

### Characteristics of the patients

A total of 80 patients were included in this study, and no patient dropped out. Table 1 presents the characteristics of the patients. There were no significant differences between the two groups.

**Table 1:** Characteristics of the patients and intraoperative data

	TMZ (n = 40)	Placebo (n = 40)	*P*
Age, years	65 ± 6	64 ± 8	0.86
Men, n (%)	28 (70.0)	26 (65.0)	0.92
BMI (Kg/m^2^)	26.3 ± 3.2	26.2 ± 3.3	0.74
*NYHA classification, n (%)*			
I	12 (30.0)	13 (32.5)	0.85
II	18 (45.0)	19 (47.5)	0.95
III	7 (17.5)	6 (15.0)	0.64
IV	3 (7.5)	2 (5.0)	0.35
*ASA classification, n (%)*			
II	6 (15.0)	7 (17.5)	0.64
III	30 (75.0)	28 (70.0)	0.76
IV	4 (10.0)	5 (12.5)	0.85
History of smoking, n (%)	12 (30)	10 (25)	0.72
History of dyslipidemia, n (%)	25 (62.5)	21 (52.5)	0.58
History of hypertension, n (%)	11 (27.5)	13 (32.5)	0.80
History of CAD, n (%)	31 (77.5)	32 (80.0)	0.86
History of percutaneous intervention or CABG for CAD, n (%)	8 (20)	9 (22.5)	0.79
History of stroke, n (%)	5 (12.5)	4 (10.0)	0.85
History of diabetes mellitus, n (%)	10 (25.0)	9 (22.5)	0.75
*Medication history, n (%)*			
ß-blockers	29 (72.5)	31 (77.5)	0.76
ACEI/ARB	30 (75.0)	32 (80.0)	0.82
Statins	26 (65.0)	27 (67.5)	0.89
Insulin	4 (10.0)	3 (7.5)	0.85
Oral anti-diabetic drugs only	10 (25.0)	11 (27.5)	0.88
Antiplatelet agents	16 (40)	14 (35)	0.74
*Type of surgery, n (%)*			
Major general	11 (27.5)	15 (37.5)	0.56
Major orthopedic	21 (52.5)	19 (47.5)	0.64
Major vascular	8 (20.0)	6 (15.0)	0.80
Preoperative troponin elevation, n (%)	2 (5.0)	3 (7.5)	0.45
Operation duration (min)	156 ± 24	162 ± 28	0.79
Blood loss (ml)	256 ± 146	267 ± 165	0.62
BIS values	45 ± 12	46 ± 14	0.83

TMZ, trimetazidine; BMI, body mass index; NYHA, New York Heart Association; ASA, American Society of Anesthesiologists; CAD, coronary artery disease; CABG, coronary artery bypass grafting; ACE, angiotensin-converting enzyme; BIS, bispectral index

### Occurrence of in-hospital cardiovascular events

**Fig. 1:**
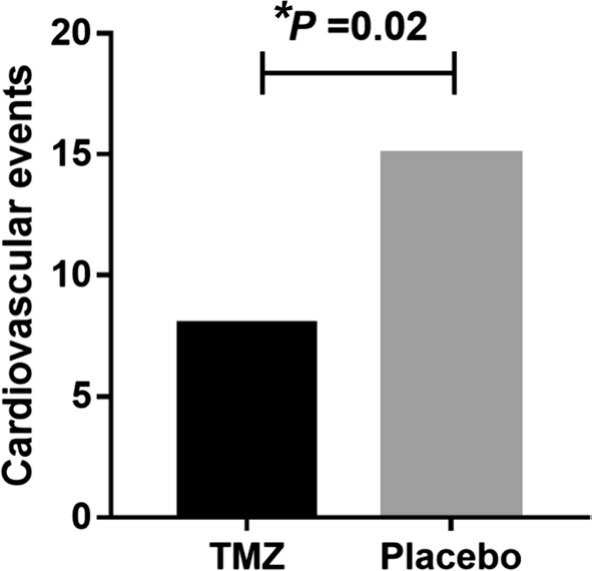
Comparison of in-hospital cardiovascular events between TMZ group and Placebo group (*P < 0.05)

**Fig. 2:**
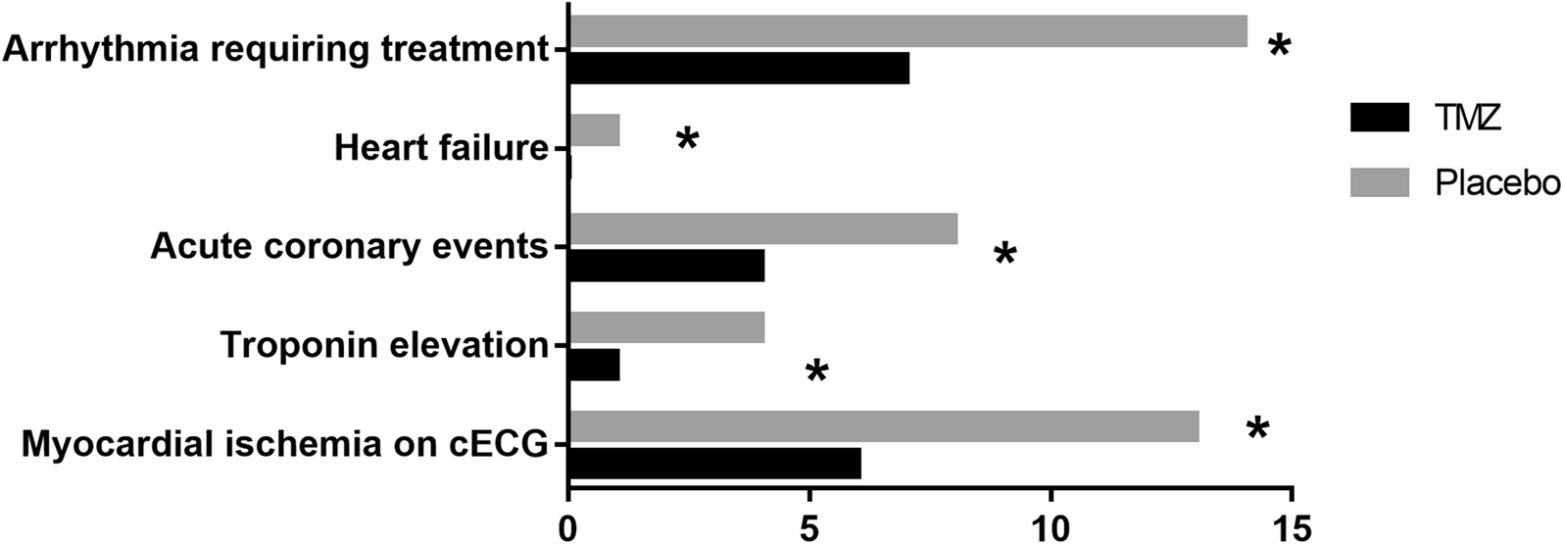
Comparison of the secondary endpoints between TMZ group and Placebo group (*P < 0.05)

**Fig. 3:**
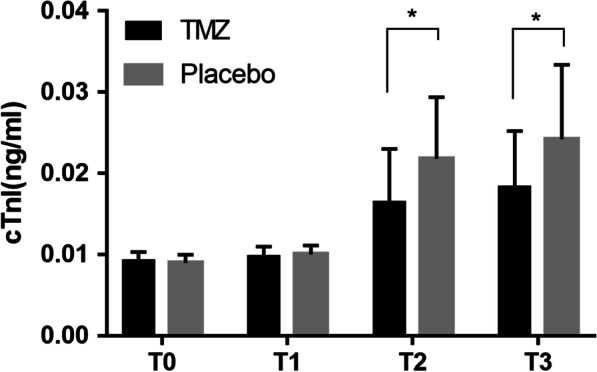
Comparison of cTnI between TMZ group and Placebo group (*P < 0.05)

Figure 1 shows that there were differences between the two groups regarding the primary endpoint (the occurrence of cardiovascular events) [8 (20.0%) vs. 15 (37.5%), *P* < 0.05]. Figure 2 indicate that the secondary endpoints were as follows: arrhythmia requiring treatment [7 (17.5%) vs. 14 (35.0%), *P* < 0.05] heart failure [0 vs. 1 (2.5%), *P* < 0.05], acute coronary event [4 (10.0%) vs. 8 (20.0%), *P* < 0.05], cTnI elevation [1 (2.5%) vs. 4 (10%), *P* < 0.01], and myocardial ischaemia [6 (15.0%) vs. 13 (32.5%), *P* < 0.01). Figure 3 shows that the cTnI level in the TMZ group was lower 24 h [0.0165 ± 0.0065 vs. 0.0218 ± 0.0076] and 48 h after surgery [0.0184 ± 0.0068 vs. 0.0242 ± 0.0092].

### Perioperative events

Table 2 shows that there were no significant differences between the two groups regarding delirium (10.0% vs. 12.5%, *P* = 0.15), incidence of postoperative nausea and vomiting (12.5% vs. 15.0%, *P* = 0.45), or length of hospital stay (*7* ± *1* vs. 9 ± 1 days, *P* = 0.20). Compared with the control group, the TMZ group had a shorter ICU stay (10 ± 2 vs. 14 ± 1 h, *P* = 0.04), but its in-hospital morbidity and mortality rates were higher (5.0% vs. 2.0%, *P* < 0.05).

**Table 2:** Postoperative data of the patients

	TMZ (n = 40)	Placebo (n = 40)	*P*
Delirium, n (%)	4 (10.0)	5 (12.5)	0.15
Incidence of postoperative nausea and vomiting, n (%)	5 (12.5)	6 (15.0)	0.45
ICU stay, hours, Mean (SD)	10 (2)	14 (1)	0.04
In-hospital morbidity and mortality, n (%)	2 (5.0)	1 (2.0)	< 0.05
Length of hospital stay, days, Mean (SD)	7 (1)	9 (1)	0.20

TMZ, Trimetazidine; ICU, intensive care unit

### Patient outcomes over 30 days

Table 3 shows the 30-day outcomes of the patients. There were no differences between the two groups regarding death (7.5% vs. 10.0%, *P* = 0.10), NYHA distribution (*P* > 0.05), cardiac dysfunction (5.0% vs. 5.0%, *P* > 0.99), or readmission (20.0% vs. 25.0%, *P* = 0.45). There was no acute myocardial infarction during the 30-day postoperative period. The occurrence of arrhythmias was lower in the TMZ group than in the control group (15.0% vs. 25.0%, *P* < 0.05).

**Table 3:** Postoperative clinical course over 30 days

	TMZ (n = 40)	Placebo (n = 40)	*P*
Death, n (%)	3 (7.5)	4 (10.0)	0.10
*NYHA, n (%)*			
I	13 (32.5)	14 (35.0)	0.65
II	19 (47.5)	18 (45.0)	0.55
III	6 (15.0)	5 (12.5)	0.45
IV	2 (5.0)	3 (7.5)	0.10
AMI, n (%)	0	0	–
Arrhythmias requiring treatment, n (%)	6 (15.0)	10 (25.0)	0.02
Cardiac dysfunction, n (%)	2 (5.0)	2 (5.0)	0.99
Readmission, n (%)	8 (20.0)	10 (25.0)	0.45

TMZ, Trimetazidine; NYHA, New York Heart Association; AMI, acute myocardial infarction

## Discussion

TMZ pretreatment protects cardiomyocytes during cardiac surgery [22, 23], but there is a lack of data on this drug in non-cardiac surgery. Therefore, the present randomized controlled trial aimed to confirm the protective effects of TMZ in elderly patients with IHD undergoing non-cardiac surgery. The results suggest that in elderly patients with IHD undergoing non-cardiac surgery, TMZ pretreatment was associated with myocardial protective effects. TMZ can be used for myocardial protection by balancing the oxygen demand and supply as well as the use of energy [21]. TMZ protects against I/R injury by improving autophagy in cardiomyocytes [12]. Previous clinical trials showed that TMZ improved myocardial functions in patients with stable coronary artery disease [19], as well as in patients undergoing CABG [22]. TMZ decreased inflammatory markers during a treadmill test [14]. In patients with diabetes undergoing percutaneous coronary intervention (PCI), TMZ decreased markers of heart and liver damage and improved heart function [24]. On the other hand, Costa et al. [25] showed that TMZ did not add any benefit to ischaemic preconditioning in patients with stable symptomatic CAD. In the present study, TMZ decreased the occurrence of in-hospital cardiovascular events (20.0% vs. 37.5%) in elderly patients with IHD undergoing non-cardiac surgery. The discrepancies among studies could be due to the selection of the patients. The present study is supported by other studies that have shown benefits from TMZ in selected patients, i.e., those with heart failure and peripheral artery disease [24], those with symptomatic stable angina [26], those undergoing PCI [22], and those with chronic heart failure [27]. Additional studies are nevertheless necessary to determine which patients might benefit the most from TMZ. As with in-hospital cardiovascular events (primary endpoint), significant differences were also observed in the secondary endpoints between the two groups, i.e., myocardial ischaemia, cTnI elevation, acute coronary event, heart failure, and arrhythmia requiring treatment. Again, this is supported by previous studies performed in a wide range of patients [24, 26]. In the present study, no TMZ-related adverse events were observed. In addition, there were no differences between the two groups regarding the occurrence of delirium or postoperative nausea and vomiting. These results suggest a good safety profile of TMZ, as reported by previous studies [28]. Major bleeding is a highly concerning risk after PCI, but the risk of haemorrhage between the two groups was not significant [29, 30].

At 30 days after surgery, there were no differences between the two groups regarding the occurrence of death, cardiac dysfunction, or readmission or the NYHA distribution. This could be because even if the patients had IHD, their IHD was not the reason for surgery, meaning that their IHD was relatively well controlled, masking potential mid-term benefits of TMZ in those patients. Nevertheless, the occurrence of arrhythmias was lower in the TMZ group, suggesting that those patients may derive some benefit from TMZ over the longer term. Additional studies are necessary to examine this point.

The present trial has limitations. First, due to the limited laboratory capabilities, we could not examine the exact molecular mechanisms of TMZ. Second, as mentioned above, there are various possible pathways by which TMZ functions as a myocardial protector. Third, this was a single-centre, small-sample trial, so the generalizability of its conclusions may be limited. Large-scale studies are needed to clarify the target population for TMZ pretreatment.

## Conclusions

In conclusion, in elderly patients with IHD undergoing non-cardiac surgery, TMZ pretreatment was associated with myocardial protective effects.

## Data Availability

All data generated or analysed during this study are included in this manuscript and its supplemental files.

## Abbreviations

TMZ: Trimetazidine
IHD: Ischaemic heart disease
cTnI: Cardiac troponin i
ASA: American society of anesthesiologists
BIS: Bispectral index
BMI: Body mass index
ECG: Electrocardiogram
AMI: Acute myocardial infarction
LOS: Length of stay
